# Supplementary material for: DIscBIO: A User-Friendly Pipeline for Biomarker Discovery in Single-Cell Transcriptomics
Source: Int J Mol Sci. 2021 Jan 30;22(3):1399. doi: 10.3390/ijms22031399 (PMC7866810; doi:10.3390/ijms22031399)
Supplement: Supplementary file 1 [file ijms-22-01399-s001.zip › Supplementary Materials/Supplementary Tables.pdf]

Supplementary Tables

DIscBIO: a user-friendly pipeline for biomarker discovery in single-cell transcriptomics

Salim Ghannoum[1*], Waldir Leoncio Netto[2], Damiano Fantini[3], Benjamin Ragan-Kelley[4], Amirabbas Parizadeh[1], Emma Jonasson[5], Anders Ståhlberg[5, 6, 7], Hesso Farhan[1] and Alvaro Köhn-Luque[2*]

[1] Department of Molecular Medicine, Institute of Basic Medical Sciences, University of Oslo, Norway.
[2] Oslo Centre for Biostatistics and Epidemiology, Faculty of Medicine, University of Oslo, Norway.
[3] Department of Urology, Northwestern University, Chicago, USA.
[4] Simula Research Laboratory, Lysaker, Norway.
[5] Sahlgrenska Center for Cancer Research, Department of Laboratory Medicine, Institute of Biomedicine, Sahlgrenska Academy at University of Gothenburg, Gothenburg, Sweden.
[6] Wallenberg Centre for Molecular and Translational Medicine, University of Gothenburg, Sweden.
[7] Department of Clinical Genetics and Genomics, Sahlgrenska University Hospital, Gothenburg, Sweden.
* Correspondence: salim.ghannoum@medisin.uio.no; alvaro.kohn-luque@medisin.uio.no; Tel.: +46 76 5770129 (S.G.)

Supplementary table S1: Networking analysis outcome of DEGs in cluster 2 of myxoid liposarcoma cells showing the genes high in both connectivity degree and betweenness centrality in addition to their known functions.

Genes	Connectivity degree	Betweenness centrality	Known Functions	References
**PLK1**	80	100.64	❖ Stemness biomarker candidate in bladder cancer.<br>❖ Affecting the chemoresistance of ovarian cancer.	[1]<br>[2]
**CDC20**	79	96.76	❖ A master cell cycle regulator<br>❖ Maintaining the self-renewal ability of prostate cancer stem cells.	[3]<br>[4]
**CDK1**	77	67.07	❖ A regulator of tumor-initiating in melanoma.	[5]
**CCNB1**	76	22.19	❖ Aactivate EMT.	[6]
**KIF2C**	76	76.55	❖ Mediating chemoresistance in ovarian cancer cells.	[7]
**KIF11**	75	28.18	❖ Critical for self-renewal and stemness in breast cancer.	[8]
**CENPE**	75	16.79	❖ Promotes sphere forming ability in glioblastoma.	[9]
**MAD2L1**	75	28.24	❖ Modulates pluripotency and stemness ability in Gastric cancer.	[10]
**CCNB2**	74	25.51	❖ Associated with cancer stem cell properties in lung squamous cell carcinoma.	[11]
**BUB1**	74	56.97	❖ Maintaining cancer stem cells in breast cancer.<br>❖ Promoting proliferation and radio-resistance in glioblastoma.	[12]<br>[13]
**AURKA**	73	3.49	❖ Involved in self-renewal of breast cancer stem cells.	[14]
**BUB1B**	73	23.07	❖ Promotes tumor proliferation and induces radioresistance in glioblastoma.	[15]
**AURKB**	73	39.61	❖ Stemness biomarker candidate in bladder cancer.	[1]
**CDCA8**	73	52.94	❖ Promoting melanoma malignant progression.	[16]
**NUF2**	72	17.88	❖ Closely linked with the progression of pancreatic cancer.	[17]
**CENPF**	72	21.95	❖ Promoting the aggressiveness of gastric cancer cells.	[18]
**CCNA2**	71	75.37	❖ A growth regulator in colorectal cancer.	[19]
**TPX2**	71	66.90	❖ Closely related to the development of breast cancer stem cells.	[20]

Supplementary references

1. Pan, S.; Zhan, Y.; Chen, X.; Wu, B.; Liu, B. Identification of biomarkers for controlling cancer stem cell characteristics in bladder cancer by network analysis of transcriptome data stemness indices. *Frontiers in oncology* **2019**, *9*, 613.
2. Chan, K.K.; Wong, O.G.W.; Wong, E.S.Y.; Chan, K.K.L.; Ip, P.P.C.; Tse, K.Y.; Cheung, A.N.Y. Impact of iASPP on chemoresistance through PLK1 and autophagy in ovarian clear cell carcinoma. *International journal of cancer* **2018**, *143*, 1456-1469.
3. Weinstein, J.; Jacobsen, F.W.; Hsu-Chen, J.; Wu, T.; Baum, L.G. A novel mammalian protein, p55CDC, present in dividing cells is associated with protein kinase activity and has homology to the Saccharomyces cerevisiae cell division cycle proteins Cdc20 and Cdc4. *Molecular and Cellular Biology* **1994**, *14*, 3350-3363.
4. Zhang, Q.; Huang, H.; Liu, A.; Li, J.; Liu, C.; Sun, B.; Chen, L.; Gao, Y.; Xu, D.; Su, C. Cell division cycle 20 (CDC20) drives prostate cancer progression via stabilization of β-catenin in cancer stem-like cells. *EBioMedicine* **2019**, *42*, 397-407.
5. Menon, D.R.; Luo, Y.; Arcaroli, J.J.; Liu, S.; KrishnanKutty, L.N.; Osborne, D.G.; Li, Y.; Samson, J.M.; Bagby, S.; Tan, A.-C. CDK1 interacts with Sox2 and promotes tumor initiation in human melanoma. *Cancer research* **2018**, *78*, 6561-6574.
6. Li, B.; Zhu, H.B.; Song, G.D.; Cheng, J.H.; Li, C.Z.; Zhang, Y.Z.; Zhao, P. Regulating the CCNB1 gene can affect cell proliferation and apoptosis in pituitary adenomas and activate epithelial-to-mesenchymal transition. *Oncology Letters* **2019**, *18*, 4651-4658.
7. Zhao, F.; Siu, M.K.; Jiang, L.; Tam, K.F.; Ngan, H.Y.; Le, X.F.; Wong, O.G.; Wong, E.S.; Gomes, A.R.; Bella, L. Overexpression of forkhead box protein M1 (FOXM1) in ovarian cancer correlates with poor patient survival and contributes to paclitaxel resistance. *PloS one* **2014**, *9*, e113478.
8. Jiang, M.; Zhuang, H.; Xia, R.; Gan, L.; Wu, Y.; Ma, J.; Sun, Y.; Zhuang, Z. KIF11 is required for proliferation and self-renewal of docetaxel resistant triple negative breast cancer cells. *Oncotarget* **2017**, *8*, 92106.
9. Behnan, J.; Grieg, Z.; Joel, M.; Ramsness, I.; Stangeland, B. Gene knockdown of CENPA reduces sphere forming ability and stemness of glioblastoma initiating cells. *Neuroepigenetics* **2016**, *7*, 6-18.
10. Pajuelo-Lozano, N.; Alcalá, S.; Sainz Jr, B.; Perona, R.; Sanchez-Perez, I. Targeting MAD2 modulates stemness and tumorigenesis in human Gastric Cancer cell lines. *Theranostics* **2020**, *10*, 9601.
11. Qin, S.; Long, X.; Zhao, Q.; Zhao, W. Co-expression network analysis identified genes associated with cancer stem cell characteristics in lung squamous cell carcinoma. *Cancer Investigation* **2020**, *38*, 13-22.
12. Han, J.Y.; Han, Y.K.; Park, G.-Y.; Kim, S.D.; Lee, C.G. Bub1 is required for maintaining cancer stem cells in breast cancer cell lines. *Scientific reports* **2015**, *5*, 1-10.
13. Yu, H.; Zhang, S.; Ibrahim, A.N.; Deng, Z.; Wang, M. Serine/threonine kinase

BUB1 promotes proliferation and radio-resistance in glioblastoma. *Pathology-Research and Practice* **2019**, *215*, 152508.
14. Yang, N.; Wang, C.; Wang, Z.; Zona, S.; Lin, S.; Wang, X.; Yan, M.; Zheng, F.; Li, S.; Xu, B. FOXM1 recruits nuclear Aurora kinase A to participate in a positive feedback loop essential for the self-renewal of breast cancer stem cells. *Oncogene* **2017**, *36*, 3428-3440.
15. Ma, Q.; Liu, Y.; Shang, L.; Yu, J.; Qu, Q. The FOXM1/BUB1B signaling pathway is essential for the tumorigenicity and radioresistance of glioblastoma. *Oncology reports* **2017**, *38*, 3367-3375.
16. Ci, C.; Tang, B.; Lyu, D.; Liu, W.; Qiang, D.; Ji, X.; Qiu, X.; Chen, L.; Ding, W. Overexpression of CDCA8 promotes the malignant progression of cutaneous melanoma and leads to poor prognosis. *International journal of molecular medicine* **2019**, *43*, 404-412.
17. Hu, P.; Shangguan, J.; Zhang, L. Downregulation of NUF2 inhibits tumor growth and induces apoptosis by regulating lncRNA AF339813. *International journal of clinical and experimental pathology* **2015**, *8*, 2638.
18. Chen, E.-B.; Qin, X.; Peng, K.; Li, Q.; Tang, C.; Wei, Y.-C.; Yu, S.; Gan, L.; Liu, T.-S. HnRNPR-CCNB1/CENPF axis contributes to gastric cancer proliferation and metastasis. *Aging (Albany NY)* **2019**, *11*, 7473.
19. Gan, Y.; Li, Y.; Li, T.; Shu, G.; Yin, G. CCNA2 acts as a novel biomarker in regulating the growth and apoptosis of colorectal cancer. *Cancer management and research* **2018**, *10*, 5113.
20. Huang, C.; Han, Z.; Wu, D. Effects of TPX2 gene on radiotherapy sensitization in breast cancer stem cells. *Oncology letters* **2017**, *14*, 1531-1535.

Supplementary table S2. A comparative analysis of DIscBIO against similar scRNAseq pipelines

	Granatum	ASAP	SC1	DIscBIO
Interface	Web-based	Web-based	Web-based	Notebook, package and web-based
Input data	csv	10x and plain-text	10x and csv	csv and Rdata
Species	Human and mouse	Human, mouse and fruit fly	Human and mouse	Any species with a taxonomy ID
Batch-effect	Yes	No	No	No
Cell filtering	Yes	Yes	Yes	Yes
Outliers detection	Yes	Yes	Yes	Yes
Gene filtering	Expression filtering (Over-dispersion)	Expression filtering	Average TF-IDF scores	Expression filtering, noise filtering (ERCC spick-ins), target gene list and combined filtering
Normalization	Quantile, Voom, geometric mean and size factor	Voom, log2, CPM, Seurat and DESeq2	-	Median of ratios (DESeq2)
Clustering	K-means, Hierarchical and Non-negative matrix factorization	K-means, Hierarchical, SNN and PAM	k-means and Hierarchical	Model based and k-means (with 9 metric measure options)
Determining the number of clusters	Automatically (elbow-point) and manually	Manually	Automatically (Gap Statistics) and manually	Automatically (Gap Statistics) and manually
Evaluating clusters	No	No	No	Jaccard, Silhouette and heatmap portrayal of cell-to-cell distances.
Data Visualization	PCA and tSNE	PCA, MDS, tSNE and ZIFA	PCA, tSNE and UMAP	PCA and tSNE
3D plots	No	Yes	Yes	No
Color-coded plots	No	Yes	Yes	Yes
Pseudo-time ordering	Yes	No	Yes	Yes
Differential Analysis (DA)	NODES, limma, edgeR and SCDE	Limma, edgeR, SCDE, DESeq2 and Voom	t-test	SAMseq and Binomial (DESeq2)
Adjustable DA parameters (FC and FDR)	No	Yes	Yes	Yes
DE between clusters	Yes	Yes	Yes	Yes
DE one vs rest	No	Yes	Yes	Yes
GEA	Yes (limited)	Yes (limited)	Yes (broad)	Yes (broad)
Protein-protein interactions	Yes	No	Yes	Yes
Networking	No	No	No	Yes
Decision trees	No	No	No	Yes

Supplementary table S3. A side-by-side evaluation of the performance of DiscBIO and Granatum using the CTC dataset

		DIscBIO	Granatum	Overlapping
Normalization		Median of ratios	Quantile normalization	
Gene filtering	Method	Based on gene expression<br>(Minexpr= 7.14 and Minnumber = 146)	Based on gene expression<br>(Log Mean Expression Threshold = 0.02 and Dispersion Fit Threshold = 0.02)	
	Outcome	209 genes were left	238 genes were left	88
Cell filtering	Method	-	-	
	Outcome	0	0	
Clustering	Method	K-means (Euclidean)	K-Means (Euclidean)	
	Outcome	4 clusters:<br>Cl1 = 26 cells<br>Cl2 = 885 cells<br>Cl3 = 451 cells<br>Cl4 = 100 cells	4 clusters:<br>Cl2 = 155 cells<br>Cl3 = 401 cells<br>Cl4 = 692 cells<br>Cl1 = 214 cells	Only 767 cells were clustered similarly (43%)
Cluster stability (Jaccard Similarities)		Cl1 = 0.43<br>Cl2 = 0.35<br>Cl3 = 0.64<br>Cl4 = 0.58<br>Average Jaccard = 0.5	Cl2 = 0.27<br>Cl3 = 0.31<br>Cl4 = 0.63<br>Cl1 = 0.61<br>Average Jaccard = 0.45	
Cluster consistency (Silhouette)		Cl1 = 0.49<br>Cl2 = 0.29<br>Cl3 = 0.40<br>Cl4 = 0.27<br>Average Silhouette = 0.33	Cl2 = 0.08<br>Cl3 = -0.29<br>Cl4 = 0.04<br>Cl1 = 0.03<br>Average Silhouette = 0.09	
Differential Analysis	Method	SAMseq<br>(fdr<0.05)	NODES<br>(Abs_z score >1.96)	
	Outcome	Cl1 vs Cl2 = 133<br>Cl1 vs Cl3 = 169<br>Cl1 vs Cl4 = 180<br>Cl2 vs Cl3 = 187<br>Cl2 vs Cl4 = 200<br>Cl3 vs Cl4 = 149	Cl2 vs Cl3 = 212<br>Cl2 vs Cl4 = 220<br>Cl2 vs Cl1 = 218<br>Cl3 vs Cl4 = 227<br>Cl1 vs Cl3 = 229<br>Cl1 vs Cl4 =211	51 DEGs<br>74 DEGs<br>71 DEGs<br>84 DEGs<br>89 DEGs<br>61 DEGs
PPI		Yes	Yes	
Pseudo-time ordering		Cells and clusters were ordered:<br>Cl1 -> Cl2 -> Cl3 -> Cl4	No Pseudo-time ordering was generated with the current settings	
